# Tricuspid Annular Abnormalities in Isolated Left Ventricular Non-compaction—Insights From the Three-dimensional Speckle-Tracking Echocardiographic MAGYAR-Path Study

**DOI:** 10.3389/fcvm.2022.694616

**Published:** 2022-05-25

**Authors:** Attila Nemes, Gergely Rácz, Árpád Kormányos

**Affiliations:** Department of Medicine, Medical Faculty, Albert Szent-Györgyi Clinical Centre, University of Szeged, Szeged, Hungary

**Keywords:** cardiomyopathy, echocardiography, three-dimensional, non-compaction, tricuspid annulus

## Abstract

**Introduction:**

Prominent trabecular left ventricular (LV) meshwork and deep intertrabecular LV recesses are featuring LV non-compaction (LVNC). The aim of this study was to evaluate tricuspid annular (TA) morphological and functional abnormalities by three-dimensional speckle-tracking echocardiography (3DSTE) in patients with LVNC without right ventricular (RV) involvement.

**Materials and Methods:**

This study consisted of 21 patients with isolated LVNC, from which 6 cases were excluded due to inferior image quality. The remaining patient group consisted of 15 subjects with a mean age of 52.1 ± 11.4 years (9 males). LVNC was defined according to the Jenni's criteria. Their results were compared to 21 age- and gender-matched healthy controls (mean age: 52.4 ± 3.9 years, 14 males). Complete routine 2-dimensional Doppler echocardiographic examination was performed in all the patients with isolated LVNC and healthy controls. End-systolic and end-diastolic TA dimensions were assessed on selected planes derived from full-volume 3D echocardiographic datasets during 3DSTE.

**Results:**

Patients with isolated LVNC showed significantly dilated end-systolic and end-diastolic TA diameter and area, which were accompanied with preserved TA functional properties and associated with right atrial (RA) volumes. TA plane systolic excursion (TAPSE) showed mild correlations with TA fractional area change (TAFAC) and TA fractional shortening (TAFS). No correlations could be demonstrated between TAPSE and TA morphological features. Extent of LVNC did not correlate with any echocardiographic parameters.

**Conclusion:**

TA is dilated with preserved sphincter-like function in patients with isolated LVNC. Longitudinal (TAPSE) and sphincter-like (TAFAC and TAFS) TA movements correlate with each other. TA dilation is associated with an increased RA volumes respecting cardiac cycle.

## Introduction

Left ventricular (LV) non-compaction (LVNC) is a rare cardiomyopathy due to arrest of the normal maturation process of the myocardium (1–3). It is characterized by prominent LV trabecularization with deep intratrabecular recesses proceeding into progressive systolic and diastolic dysfunction, conduction abnormalities, and thromboembolic events. In most of the cases, LVNC is isolated, but biventricular forms are also known (4). LVNC can be accompanied with significant valvular functional regurgitations due to dilation of heart chambers (5–7). Although it was recognized as a separate clinical entity more than 40 years ago, still several clinical questions remained unsolved (1).

The tricuspid valve (TV) is a complex anatomical structure, which incorporates the three-dimensional (3D) saddle-shaped annulus fibrosus [tricuspid annulus (TA)] (6). 3D speckle-tracking echocardiography (STE) is a novel non-invasive imaging method offering not only volumetric, strain, and rotational assessment of heart chambers, but with finding optimal valvular planes in the 3D space measurement of TA dimensions respecting cardiac cycle is also allowed (8–11). Although TA is a fibrous ring, it is also strongly connected to the adjacent muscle tissues; therefore, any changes with muscle compaction during embryogenesis could be theorized to be associated with valvular annular abnormalities. This study was designed to assess TA morphological and functional abnormalities by 3DSTE in patients with LVNC without right ventricular (RV) involvement.

## Materials and Methods

### Patient Population

**Figure 1:**
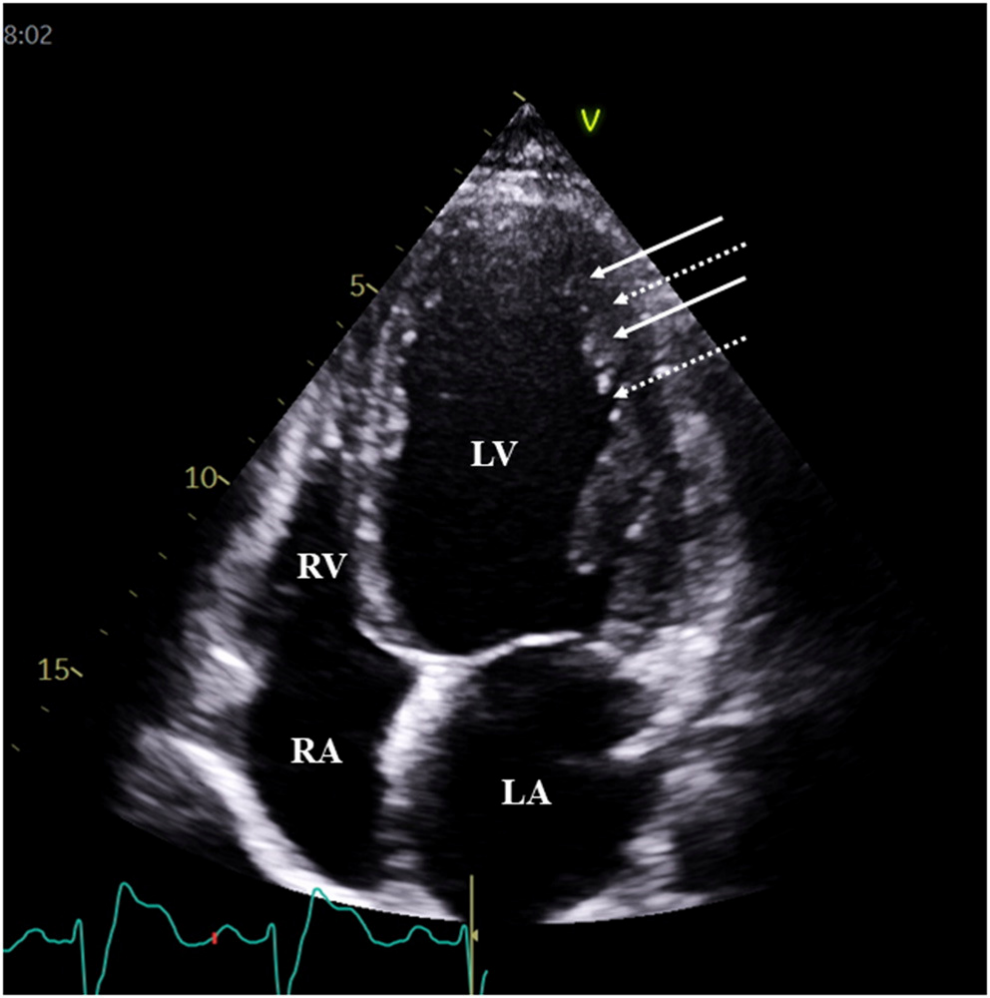
Typical two-dimensional echocardiographic apical four-chamber view demonstrating prominent trabeculations (white arrows) and intertrabecular recesses (dashed arrows) in a patient with typical features of left ventricular non-compaction. White arrows represent trabeculae, while dotted arrows represent sinusoids.

This study comprised patients with isolated LVNC, which was defined according to the Jenni's criteria (Figure 1) (5):

- An excessively thickened LV myocardial wall with a 2-layered structure comprising a compacted layer on the epicardial side and a non-compacted layer of prominent trabeculations and deep intertrabecular recesses on the endocardial side.
- A non-compacted/compacted LV myocardium thickness ratio > 2 measured at the moment of maximal thickness in end-systole.
- Color Doppler evidence of deep intertrabecular recesses in communication with the LV cavity.
- Absence of coexisting cardiac anomalies.

Their results were compared to age- and gender-matched healthy controls who were considered to be healthy in the absence of any disorder, other pathological state or risk factor, regular drug use, and negative electrocardiographic and echocardiographic findings. Complete routine 2-dimensional (2D) Doppler echocardiographic examination was performed in all the patients with isolated LVNC and healthy controls. This study is a part of the **M**otion **A**nalysis of the heart and **G**reat vessels b**Y** three-dimension**A**l speckle-t**R**acking echocardiography in **Path**ological cases **(MAGYAR-Path) Study**, which aimed to examine valvular annular abnormalities in different disorders among other aims (“Magyar” means “Hungarian” in hungarian language). This methodology complied with the Declaration of Helsinki, informed consent was obtained from all the patients and healthy controls, and the institutional review board approved it (registration number: 71/2011).

### Two-Dimensional Doppler Echocardiography

**Figure 2:**
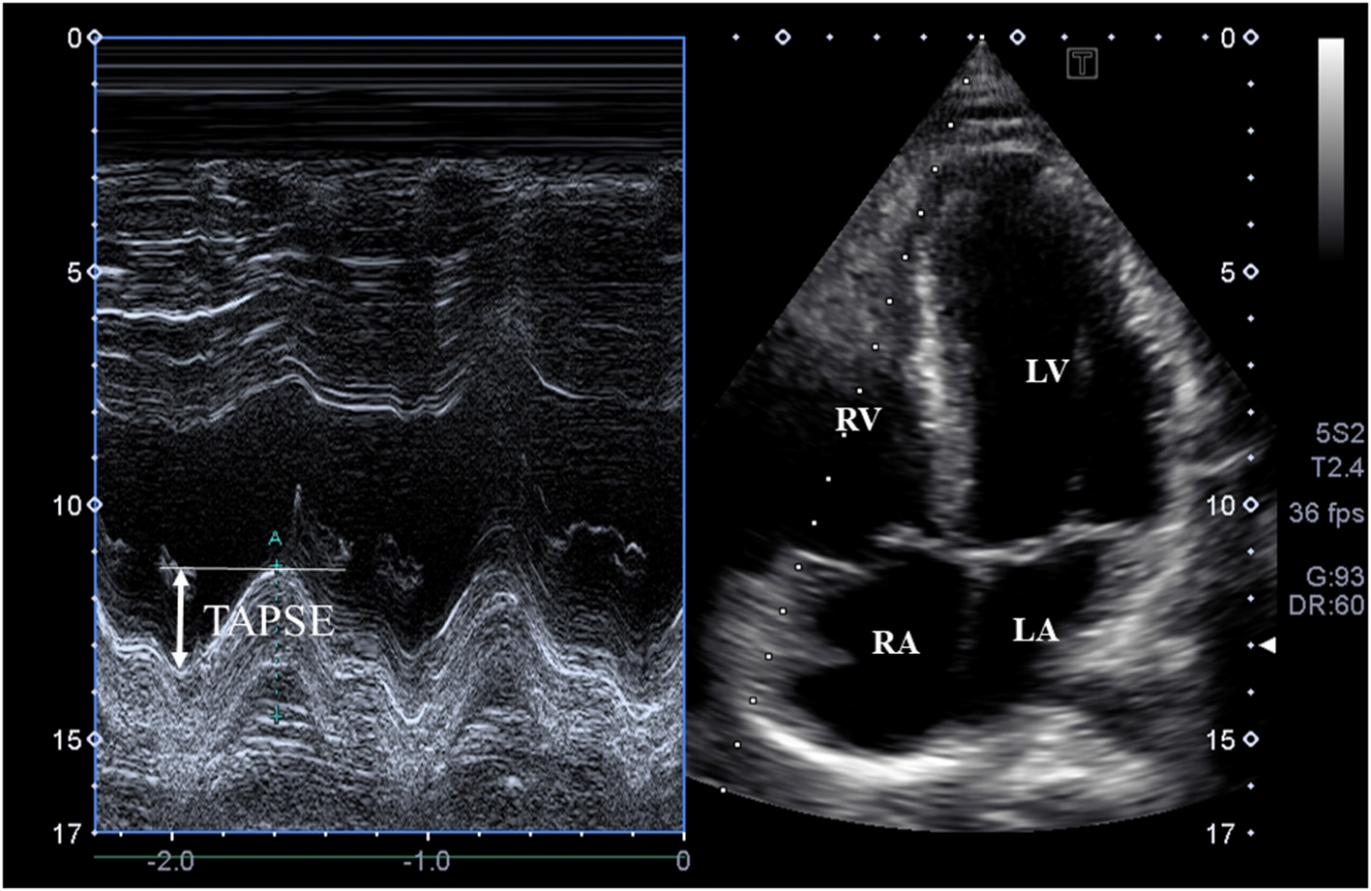
M-mode echocardiography-derived assessment of tricuspid annular plane systolic excursion (TAPSE) on apical four-chamber view.

Standard 2D Doppler echocardiographic examination included measurement of 2D echocardiography-derived LV dimensions, volumes and ejection fraction, left atrial diameter from parasternal view, Doppler featuring of LV diastolic function by measurement of transmitral E and A waves, and valvular gradients and visual color Doppler characterization of valvular regurgitations. TA plane systolic excursion (TAPSE) (Figure 2) and right ventricular fractional area change (RVFAC) were also calculated (7, 12). The extent of LVNC was characterized by the number of non-compacted segments using the 16-segment LV model. For these aims, commercially available Toshiba Artida^TM^ echocardiographic machine (Toshiba Medical Systems, Tokyo, Japan) attached to a PST-30SBP (1–5 MHz) phased-array transducer was used (12).

### Three-Dimensional STE

During 3DSTE, the same Toshiba Artida^TM^ echocardiographic machine (Toshiba Medical Systems, Tokyo, Japan) with a 1–4 MHz matrix phased-array PST-25SX transducer was used. Data acquisitions were performed within a single breath-hold and during a constant RR interval. In all the cases, 6 wedge-shaped subvolumes were acquired and then the software created a full volumes pyramid-shaped 3D dataset, where the analysis and chamber quantification were done later (8–10).

### Three-Dimensional Speckle-Tracking Echocardiography-Derived Tricuspid Annular Measurements

**Figure 3:**
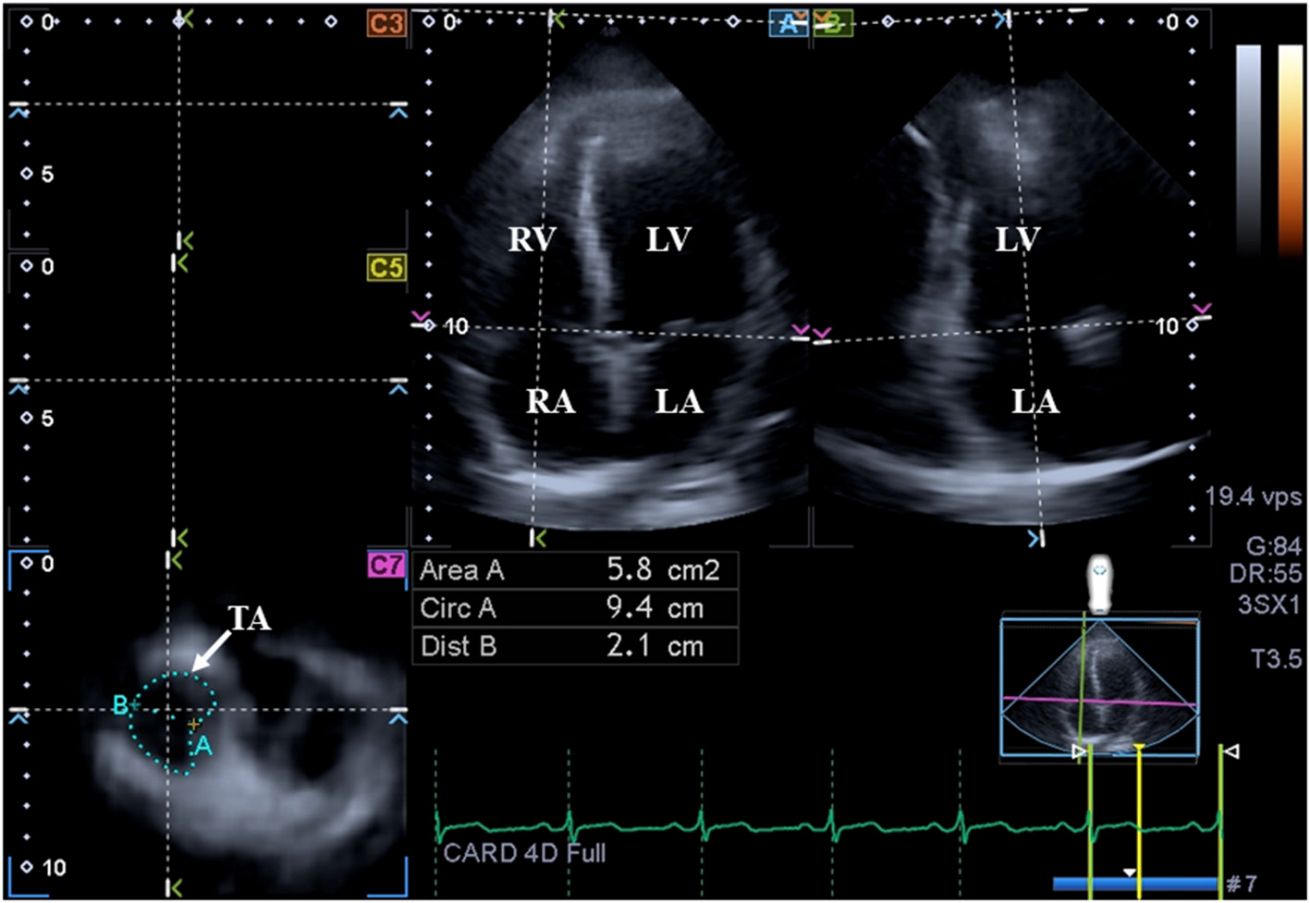
Assessment of the tricuspid annulus is presented extracted from a three-dimensional full-volume dataset: apical four-chamber view (A); apical two-chamber view (B); and a cross-sectional view at the level of the tricuspid annulus optimized in apical four- and two-chamber views (C7). The white arrow represents the tricuspid annular plane. LA, left atrium; LV, left ventricle; TA, tricuspid annulus; RA, right atrium; RV, right ventricle; Area, TA area; Circ, TA perimeter; Dist, TA diameter.

Tricuspid annulus was examined according to the 3D echocardiography-derived methodology presented previously in more details by the 3D Wall Motion Tracking software version 2.7 (Toshiba Medical Systems, Tokyo, Japan) (11). Shortly, following finding optimal non-foreshortened long-axis LV views using the acquired 3D dataset, plane of TA was defined on apical two-chamber (AP2CH) and apical four-chamber (AP4CH) views and then “en-face” TA view was created on C7 short-axis view. End-diastolic (just before tricuspid valve closure) and end-systolic (just before tricuspid valve opening) assessments were performed for morphological and functional TA parameters (Figure 3).

#### Morphological Parameters

- Tricuspid annular diameter (TAD), defined as the perpendicular line drawn from the peak of TA curvature to the middle of the straight TA border.
- Tricuspid annular area (TAA), measured by planimetry.
- Tricuspid annular perimeter (TAP), measured by planimetry.

#### Functional Parameters

- Tricuspid annular fractional shortening (TAFS) = [(end-diastolic TAD - end-systolic TAD)/end-diastolic TAD] × 100.
- Tricuspid annular fractional area change (TAFAC) = [(end-diastolic TAA - end-systolic TAA)/end-diastolic TAA] × 100.

### Three-Dimensional Speckle-Tracking Echocardiography-Derived Right Atrial (RA) Assessments

**Figure 4:**
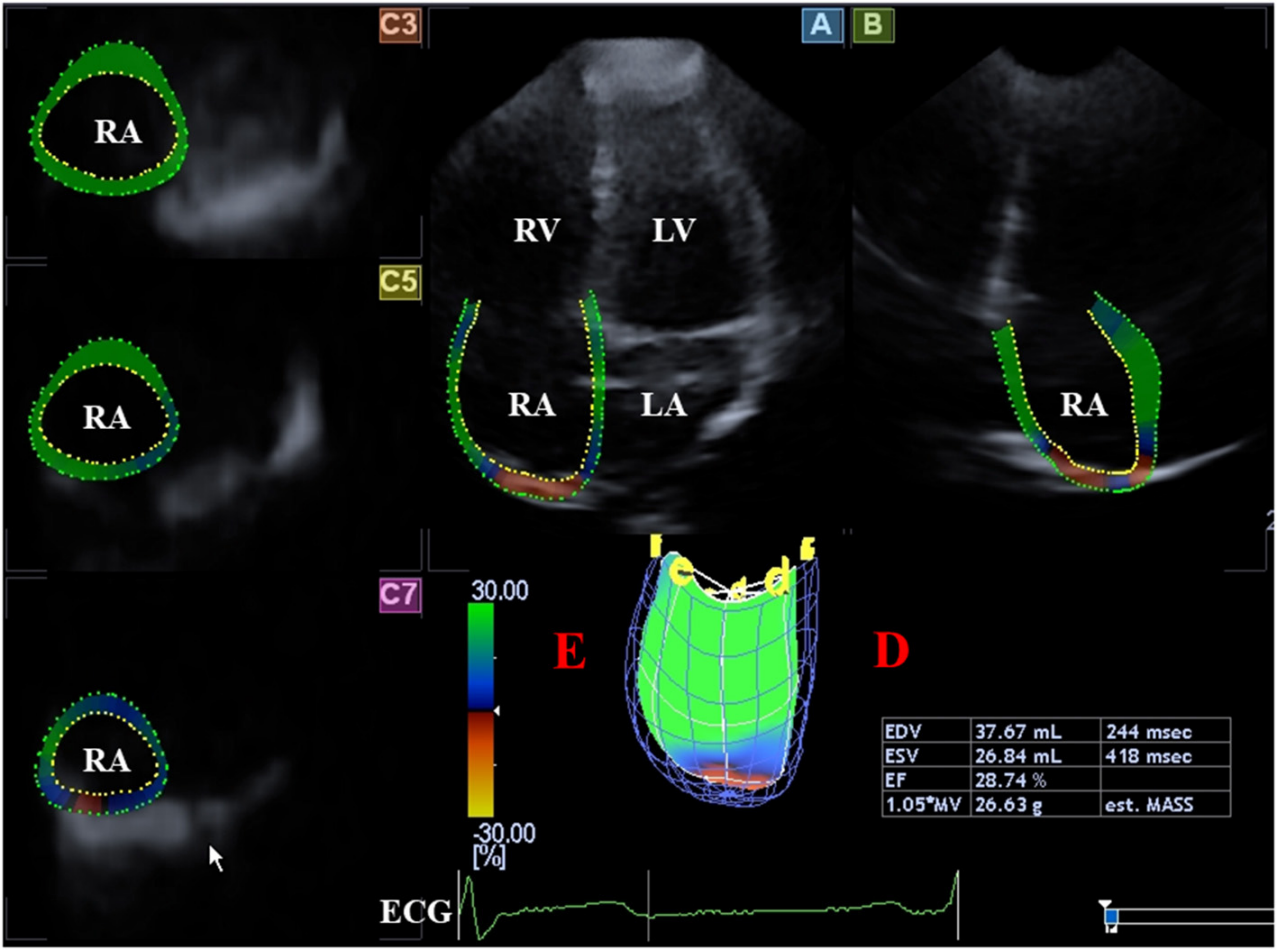
Evaluation of the right atrium is presented extracted from a three-dimensional speckle-tracking echocardiography-derived full-volume dataset: Apical four-chamber (A) and two-chamber (B) views and parasternal short-axis views at basal (C3), midatrial (C5),; and superior (C7) right atrial level are demonstrated. Right atrial volumetric data (D) and virtual 3D three-dimensional right atrial model (E) are also presented. ECG, electrocardiography; EDV, end-diastolic volume; ESV, end-systolic volume, EF, ejection fraction; RA, right atrium; RV, right ventricle.

The same software was used for virtual 3D model creation and quantification of the right atrium (RA) (13). First, AP2CH and AP4CH views and three short-axis views on different levels of the RA were created at end-diastole. Following detection of optimal non-foreshortened views on long-axis views, markers were set starting from the edge of septum-tricuspid ring in a clockwise way around the RA to the edge of the lateral wall-tricuspid ring. RA appendage, caval veins, and coronary sinus were excluded from the assessments. At the end, the software automatically reconstructed 3D surface of the RA during the cardiac cycle creating a virtual 3D cast of the RA (Figure 4). Using this 3D RA model, end-systolic maximum, early diastolic preatrial contraction, and late diastolic minimum RA volumes were calculated respecting the cardiac cycle together with longitudinal, circumferential and radial peak RA strains representing systolic RA reservoir function.

### Statistical Analysis

Calculated data were demonstrated as mean ± SD or absolute numbers/percentages as appropriate. Homogeneity of variances were assessed by Levene's test. If a dataset proved to be normally distributed, the Student's *t*-test was used, while if it was found non-normally distributed, the Mann–Whitney *U* test or the Wilcoxon signed-rank test was performed. The chi-squared test/Fisher's exact test was used for statistical analysis as appropriate. Correlations were established by calculation of Pearson's correlation coefficients. They were used for intraobserver and interobserver correlations as well. Intraobserver and interobserver agreements were evaluated using the Bland–Altman method. All the tests were two-sided. *p* < 0.05 was defined as statistical significant. The statistical package used was SPSS software version 25.1.

## Results

### Clinical Characteristics

This study consisted of 21 patients with isolated LVNC, from which 6 cases were excluded due to inferior image quality. The remaining patient group consisted of 15 subjects with a mean age of 52.1 ± 11.4 years (9 males). Although RV is more trabeculated in normal circumstances than the LV, none of the cases showed obvious RV hypertrabeculation and typical signs of NC. The control group consisted of 21 age- and gender-matched healthy subjects (mean age: 52.4 ± 3.9 years, 14 males). Body surface area of healthy controls and patients with isolated LVNC proved to be 1.82 ± 0.23 and 1.82 ± 0.34 m^2^, respectively. Clinical data of patients with isolated LVNC and healthy controls are given in Table 1.

**Table 1:** Baseline demographic and two-dimensional echocardiographic data of patients with isolated left ventricular non-compaction and controls.

	**Controls** **(*n* = 21)**	**Isolated** **LVNC** **patients** **(*n* = 15)**
**Risk factors**		
Age (years)	52.4 ±3.9	52.1 ± 11.4
Male gender (%)	14 (67)	9 (60)
Hypertension (%)	0 (0)	6 (40)^*^
Diabetes mellitus (%)	0 (0)	0 (0)
Hypercholesterolaemia (%)	0 (0)	3 (20)
**Two-dimensional echocardiography**		
LA diameter (mm)	38.2 ± 5.5	44.6 ± 7.8^*^
LV end-diastolic diameter (mm)	47.6 ± 6.1	61.2 ± 11.8^*^
LV end-diastolic diameter-indexed (mm/m^2^)	26.2 ± 3.0	33.5 ± 6.4
LV end-diastolic volume (mL)	107.5 ± 19.5	186.4 ± 84.8^*^
LV end-diastolic volume-indexed (mL/m^2^)	59.1 ± 9.6	102.5 ± 48.4
LV end-systolic diameter (mm)	31.6 ± 3.7	46.2 ± 14.4^*^
LV end-systolic diameter-indexed (mm/m^2^)	17.5 ± 1.8	25.4 ± 7.8
LV end-systolic volume (mL)	35.7 ± 9.0	106.4 ± 73.2^*^
LV end-systolic volume-indexed (mL/m^2^)	19.7 ± 4.6	58.5 ± 37.4
Interventricular septum (mm)	9.5 ± 1.4	9.9 ± 1.4
LV posterior wall (mm)	9.9 ± 1.5	9.7 ± 1.2
LV ejection fraction (%)	66.3 ± 3.2	42.4 ± 14.4^*^

*LVNC, left ventricular non-compaction; LA, left atrial; LV, left ventricular*.

* *p < 0.05 vs. Controls*.

### Two-Dimensional Doppler Echocardiographic Data

Standard 2D echocardiographic data are shown in Table 1. Enlarged left atrial (LA) and LV dimensions with reduced LV ejection fraction could be seen in patients with isolated LVNC as compared to that of matched controls. Indexed RV end-diastolic short-axis diameter and area were 16.5 ± 2.4 mm/m^2^ and 9.8 ± 2.3 cm^2^/m^2^ in patients with isolated LVNC, respectively. The mean number of non-compacted segments proved to be 6.9 ± 2.0. Grade 1 and 2 mitral regurgitation (MR) could be demonstratred in 5 and 5 patients with isolated LVNC, respectively. Higher grade of MR could not be found. Grade 1 and 2 tricuspid regurgitation (TR) could be demonstrated in 4 and 1 patients with isolated LVNC, respectively. Only one isolated patient with LVNC showed grade 4 TR. None of patients with isolated LVNC and healthy controls showed significant valvular stenosis. TAPSE and RVFAC proved to be 13.9 ± 3.9 mm and 34.1 ± 2.7%, respectively, in patients with isolated LVNC.

### Three-Dimensional Speckle-Tracking Echocardiographic Data

Patients with isolated LVNC showed significantly dilated end-systolic and end-diastolic TA diameter and area, which were accompanied with preserved TA functional properties. RA volumes respecting cardiac cycle proved to be dilated in patients with isolated LVNC (Table 2).

**Table 2:** Comparison of three-dimensional speckle-tracking echocardiography-derived tricuspid annular morphological and functional parameters and right atrial volumes between patients with isolated left ventricular non-compaction and controls.

	**Controls** **(*n* = 21)**	**Isolated** **LVNC** **patients** **(*n* = 15)**
**Morphologic tricuspid annular parameters**		
TAD-D (cm)	2.2 ± 0.3	2.6 ± 0.3^*^
TAA-D (cm^2^)	7.0 ± 1.5	8.4 ± 1.7^*^
TAP-D (cm)	10.5 ± 1.2	11.2 ± 1.4
TAD-S (cm)	1.8 ± 0.3	2.2 ± 0.2^*^
TAA-S (cm^2^)	5.3 ± 1.4	6.5 ± 1.7^*^
TAP-S (cm)	8.9 ± 1.0	9.8 ± 1.5
**Functional tricuspid annular parameters**		
TAFAC (%)	23.7 ± 11.7	22.2 ± 12.3
TAFS (%)	18.8 ± 8.0	15.9 ± 5.6
**RA volumes and global peak strains**		
V_max_ (ml)	47.3 ± 9.8	56.6 ± 15.3^*^
V_max_ - indexed (ml/m^2^)	25.8 ± 4.8	31.1 ± 8.4^*^
V_preA_ (ml)	37.7 ± 9.9	48.0 ± 14.4^*^
V_preA_ - indexed (ml/m^2^)	20.7 ± 4.9	26.4 ± 8.3^*^
V_min_ (ml)	29.3 ± 10.1	39.6 ± 15.5^*^
V_min_ - indexed (ml/m^2^)	16.1 ± 5.0	21.8 ± 8.7^*^
LS (%)	22.5 ± 10.5	22.7 ± 9.8
CS (%)	10.2 ± 7.5	13.0 ± 13.1
RS (%)	−14.5 ± 8.6	−9.8 ± 8.2

*D, end-diastolic; S, end-systolic; LVNC, left ventricular non-compaction; CS, circumferential strain; LS, longitudinal strain; RS, radial strain; TAD, tricuspid annular diameter; TAA, tricuspid annular area; TAP, tricuspid annular perimeter; TAFAC, tricuspid annular fractional area change; TAFS, tricuspid annular fractional shortening; V_max_, end-systolic maximum RA volume; V_preA_, early diastolic RA volume before atrial contraction; V_min_, end-diastolic minimum RA volume*.

* *p < 0.05 vs. Controls*.

### Correlations

Tricsupid annular plane systolic excursion and RVFAC showed mild correlations with TAFAC (*r* = 0.39, *p* = 0.05; *r* = 0.36, *p* = 0.05, respectively) and TAFS (*r* = 0.37, *p* = 0.05; *r* = 0.38, *p* = 0.05). No correlations could be demonstrated between TAPSE and RVFAC and TA morphological parameters. RA strains did not correlate with any TA morphological and functional features. Extent of LVNC did not correlate with any echocardiographic parameters. End-diastolic and end-systolic TAD and TAA correlated with all the RA volumes, while end-diastolic and end-systolic TAP showed correlations with V_min_ only. Only TAFS correlated with V_min_, other correlations between TA functional properties, and RA volumes could not be detected (Table 3).

**Table 3:** Correlation coefficients of right atrial volumes and tricuspid annular morphological and functional parameters in patients with isolated left ventricular non-compaction.

	**V_**max**_**	**V_**preA**_**	**V_**min**_**
TAD-D	0.60^*^	0.48^*^	0.74^*^
TAA-D	0.66^*^	0.58^*^	0.77^*^
TAP-D	0.24	0.23	0.54^*^
TAD-S	0.59^*^	0.57^*^	0.79^*^
TAA-S	0.62^*^	0.53^*^	0.71^*^
TAP-S	0.27	0.34	0.64^*^
TAFAC	−0.09	−0.21	−0.17
TAFS	−0.16	−0.30	−0.42^*^

*D, end-diastolic; S, end-systolic; TA, tricuspid annulus; TAD, tricuspid annular diameter; TAA, tricuspid annular area; TAP, tricuspid annular perimeter; TAFAC, tricuspid annular fractional area change; TAFS, tricuspid annular fractional shortening; V_max_, end-systolic maximum RA volume; V_preA_, early diastolic RA volume before atrial contraction; V_min_, end-diastolic minimum RA volume*.

* *p < 0.05*.

### Reproducibility of 3DSTE-Derived TA Measurements

The mean ± SD difference in values of 3DSTE-derived end-diastolic and end-systolic TA diameter, area, and perimeter measured two times by the same observer and by two independent observers, along with the respective correlation coefficients are given in Table 4.

**Table 4:** Intra- and interobserver variability for three-dimensional speckle-tracking echocardiographic measurement of tricuspid annular dimensions.

	**Intraobserver agreement**		**Interobserver agreement**	
	**mean ± 2SD difference in values obtained by 2 measurements of the same observer**	**correlation coefficient between measurements of the same observer**	**mean ± 2SD difference in values obtained by 2 observers**	**correlation coefficient between independent measurements of 2 observers**
End-diastolic TAD	0.02 ± 0.25 cm	0.95 (p <0.0001)	0.04 ± 0.20 cm	0.97 (p <0.0001)
End-diastolic TAA	−0.03 ± 1.21 cm^2^	0.95 (p <0.0001)	0.02 ± 0.68 cm^2^	0.97 (p <0.0001)
End-diastolic TAP	−0.04 ± 0.76 cm	0.96 (p <0.0001)	−0.13 ± 0.64 cm	0.96 (p <0.0001)
End-systolic TAD	−0.04 ± 0.19 cm	0.97 (p <0.0001)	0.02 ± 0.45 cm	0.97 (p <0.0001)
End-systolic TAA	−0.04 ± 0.44 cm^2^	0.96 (p <0.0001)	−0.06 ± 0.71 cm^2^	0.95 (p <0.0001)
End-systolic TAP	0.08 ± 0.62 cm	0.98 (p <0.0001)	0.04 ± 0.65 cm	0.98 (p <0.0001)

*TAD, tricuspid annular diameter; TAA, tricuspid annular area; TAP, tricuspid annular perimeter; SD, standard deviation*.

### Feasibility of 3DSTE-Derived TA Measurements

Six out of 21 patients with LVNC (29%) and 9 out of 30 healthy subjects (30%) were excluded due to insufficient image quality (poor quality for visual qualitative analysis with or without artifacts). The overall feasibility of measurements proved to be 70%.

## Discussion

Tricuspid annulus has a complex, asymmetrical, and saddle-shaped ellipsoid structure with a dynamic nature respecting cardiac cycle. Several echocardiographic methods can be used for morphological and functional assessment of the TA including simple M-mode echocardiography-derived TAPSE, which featuring its systolic longitudinal movement along long-axis of the RV (7). TAPSE is a relatively load- and angle-dependent parameter with a significant prognostic impact. TAFAC and TAFS are based on 3DSTE-derived end-diastolic and end-systolic TA diameter and area and characterize sphincter-like TA movements (11). Using these TA functional parameters together can give us an opportunity for complex spatial TA assessment. It can be important especially in disorders with potential functional valvular regurgitations such as LVNC (14). Functional TR is considered to be a multifactorial disorder, resulting from maladaptive RV remodeling leading to RA, TA, and basal RV dilation with pathological TV coaptation (14). It has been confirmed that TR is highly dependent on TA dilation (6).

Isolated LVNC was found to be associated with significant abnormalities in myocardial mechanics in several studies. Systolic LV dysfunction did not confine to the non-compacted segments in isolated LVNC, non-compacted and compacted LV segments have been demonstrated to have comparably increased regional LV volumes, reduced regional LV ejection fractions, and decreased LV strains demonstrating deteriorated LV function (15, 16). LV radial and 3D strains showed further reduction in non-compacted segments compared to compacted segments (16). In most patients with LVNC, absence of LV twist could be detected when LV base and apex rotating in the same clockwise or counterclockwise direction. This clinical situation is called LV “rigid body rotation” (17, 18). Significant LV abnormalities were associated with increased all the LA volumes and reduced LA emptying fractions (EFs) with preserved LA stroke volumes (SVs) respecting cardiac cycle in LVNC (19). Moreover, peak LA strains representing its systolic function proved to be impaired (19), while LA ejection force representing its late diastolic function proved to be enhanced (20). Most of LA abnormalities were related to future events (19), but LA and LV abnormalities were not related to the extent of LVNC (15, 16, 19). All these changes in left heart chamber volumes and functional properties were associated with dilation of mitral annular dimensions and functional impairment, which could partially explain LVNC-related functional MRs (21).

According to these abnormalities accompanied with LVNC, there can be a question, what happens with the right heart in isolated form of LVNC. In a recent study, increased RA volumes respecting heart cycle could be demonstrated (13). In contrast with LA abnormalities, only passive RA-SV proved to be reduced, while remaining RA-SVs and RA-EFs were found to be preserved. Although RV myocardium displays more trabeculations in LVNC, end-diastolic RV size is increased with decreased systolic RV function, which is predictor of adverse outcome in patients with LVNC (22).

There are little information on the tricuspid valve in LVNC and most of them are case reports focusing on associated TV abnormalities (23, 24). In this study, TA dilation could be demonstrated in a series of patients with isolated LVNC without significant TR (except 1 case), which was associated with increased RA volumes. Although TAPSE representing longitudinal movement of the TA along its long axis was lower than normal, TAFAC and TAFS did not differ significantly between patients with isolated LVNC and healthy controls suggesting preserved sphincter-like TA motion. Longitudinal- and sphincter-like TA movements correlated with each other. The results could suggest that although there is a significant myocardial disease with severe dilation of heart chambers, no deterioration of TA sphincter-like function with consecutive significant TR could be detected. Although LV is dysfunctional, RA dilation is not associated with significant impairment of RA strains in patients with isolated LVNC (13). However, RA strains did not correlate with TA features. It could be theoretized that by losing TA sphincter-like function, TR will develop. All the changes detailed above could be explained by isolated LVNC-associated interventricular and interatrial interactions, changes in loading and pressure conditions, muscle wall and annular tissue abnormalities, etc. However, further studies area warranted to confirm our findings, especially focusing on their diagnostic and prognostic value. Theoretically, compensatory RV global or regional RV hyperfunction could elucidate preserved TA sphincter-like motion, but should be confirmed by more recent and advanced imaging techniques such as echocardiography-derived RV strains or MRI.

## Limitations

- Image quality of 3DSTE is lower than that of 2D echocardiography, which could affect measurements (8–10).
- Only clear patients with LVNC were evaluated and biventricular form of NC or unclear cases with extensive RV hypertrabecularization was excluded from this study.
- Only a limited number of patients with LVNC were assessed. However, it could be considered that LVNC is a rare disease and results origin from a single center (1).
- Some patients with LVNC showed small grade of TA regurgitation. Moreover, from risk factors, hypertension and hypercholesterolemia were relatively frequent in the group of patients with isolated LVNC, which could affect results.
- The normal ranges of 3DSTE-derived TA parameters are missing from the literature; therefore, comparison of TA parameters of healthy control subjects to these data is not possible at this moment.
- End-systolic and end-diastolic TA dimensions were given for all the controls and patients with isolated LVNC. Other parameter of TA was not aimed to be measured during evaluations.
- Comparison results of patients with LVNC with that of a control population of patients with dilated cardiomyopathy (DCM) without LVNC would have improved understanding the findings. However, involvement of patients with DCM was not possible at the time of echocardiographic examinations.
- Tricuspid regurgitation was quantified by visual assessment. More advanced TR quantification system was not applied in this study.

## Conclusion

Tricuspid annulus is dilated with preserved sphincter-like function in patients with isolated LVNC. Longitudinal (TAPSE) and sphincter-like (TAFAC and TAFS) TA movements correlate with each other. TA dilation is associated with an increased RA volumes respecting cardiac cycle.

## Data Availability Statement

The original contributions presented in the study are included in the article/supplementary materials, further inquiries can be directed to the corresponding authors.

## Ethics Statement

The studies involving human participants were reviewed and approved by Institutional Review Board at the University of Szeged (Registration Number: 71/2011). The patients/participants provided their written informed consent to participate in this study.

## Author Contributions

AN: hypothesis and writing paper. ÁK: performing 3DSTE, measurements, and statistics. GR: performing measurements and statistics. All authors contributed to the article and approved the submitted version.

## Funding

This work was supported by University of Szeged Open Access Fund.

## Conflict of Interest

The authors declare that the research was conducted in the absence of any commercial or financial relationships that could be construed as a potential conflict of interest.

## Publisher's Note

All claims expressed in this article are solely those of the authors and do not necessarily represent those of their affiliated organizations, or those of the publisher, the editors and the reviewers. Any product that may be evaluated in this article, or claim that may be made by its manufacturer, is not guaranteed or endorsed by the publisher.

## References

[1] Engberding R Bender F. Identification of a rare congenital anomaly of the myocardium by two-dimensional echocardiography: persistence of isolated myocardial sinusoids. Am J Cardiol. (1984) 53:1733–4. 10.1016/0002-9149(84)90618-0 6731322

[2] Filho DCS do Rêgo Aquino PL de Souza Silva G Fabro CB. Left ventricular non-compaction: new insights into a poorly understood disease. Curr Cardiol Rev. (2021) 17:209–16. 10.2174/1573403X16666200716151015 32674738 PMC8226207

[3] Rao K Bhaskaran A Choudhary P Tan TC. The role of multimodality imaging in the diagnosis of left ventricular non-compaction. Eur J Clin Invest. (2020) 50:e13254. 10.1111/eci.13254 32329049

[4] Nemes A Caliskan K Soliman OI McGhie JS Geleijnse ML ten Cate FJ. Diagnosis of biventricular non-compaction cardiomyopathy by real-time three-dimensional echocardiography. Eur J Echocardiogr. (2009) 10:356–7. 10.1093/ejechocard/jep008 19213803

[5] Jenni R Oechslin E Schneider J Attenhofer Jost C Kaufmann JA. Echocardiographic and pathoanatomical characteristics of isolated left ventricular non-compaction: a step towards classification as a distinct cardiomyopathy. Heart. (2001) 86:666–71. 10.1136/heart.86.6.666 11711464 PMC1730012

[6] Yucel E Bertrand PB Churchill JL Namasivayam M. The tricuspid valve in review: anatomy, pathophysiology and echocardiographic assessment with focus on functional tricuspid regurgitation. J Thorac Dis. (2020) 12:2945–54. 10.21037/jtd.2020.02.42 32642207 PMC7330354

[7] Rudski LG Lai WW Afilalo J Hua L Handschumacher MD Chandrasekaran K. Guidelines for the echocardiographic assessment of the right heart in adults: a report from the American Society of Echocardiography endorsed by the European Association of Echocardiography, a registered branch of the European Society of Cardiology, and the Canadian Society of Echocardiography. J Am Soc Echocardiogr. (2010) 23:685–713. 10.1016/j.echo.2010.05.010 20620859

[8] Urbano-Moral JA Patel AR Maron MS Arias-Godinez JA Pandian NG. Three-dimensional speckle tracking echocardiography: Methodological aspects and clinical potential. Echocardiography. (2012) 29:997–1010. 10.1111/j.1540-8175.2012.01773.x 22783969

[9] Ammar KA Paterick TE Khandheria BK Jan MF Kramer C Umland MM. Myocardial mechanics: understanding and applying three-dimensional speckle tracking echocardiography in clinical practice. Echocardiography. (2012) 29:861–72. 10.1111/j.1540-8175.2012.01712.x 22591237

[10] Nabeshima Y Seo Y Takeuchi M. A review of current trends in three-dimensional analysis of left ventricular myocardial strain. Cardiovasc Ultrasound. (2020) 18:23. 10.1186/s12947-020-00204-3 32591001 PMC7320541

[11] Anwar AM Soliman OI Nemes A van Geuns RJ Geleijnse ML Ten Cate FJ. Value of assessment of tricuspid annulus: real-time three-dimensional echocardiography and magnetic resonance imaging. Int J Cardiovasc Imaging. (2007) 23:701–5. 10.1007/s10554-006-9206-4 17295104 PMC2048828

[12] Lang RM Badano LP Mor-Avi V Afilalo J Armstrong A Ernande L. Recommendations for cardiac chamber quantification by echocardiography in adults: an update from the American Society of Echocardiography and the European Association of Cardiovascular Imaging. Eur Heart J Cardiovasc Imaging. (2015) 16:233–70. 10.1093/ehjci/jev014 25712077

[13] Nemes A Domsik P Kalapos A Gavallér H Oszlánczi M Forster T. Right atrial deformation analysis in isolated left ventricular non-compaction - insights from the three-dimensional speckle tracking echocardiographic MAGYAR-Path Study. Rev Port Cardiol. (2016) 35:515–21. 10.1016/j.repc.2016.04.009 27609553

[14] Condello F Gitto M Stefanini GG. Etiology, epidemiology, pathophysiology and management of tricuspid regurgitation: an overview. Rev Cardiovasc Med. (2021) 22:1115–42. 10.31083/j.rcm2204122 34957757

[15] Nemes A Caliskan K Geleijnse ML Soliman OI Vletter WB ten Cate FJ. Reduced regional systolic function is not confined to the non-compacted segments in non-compaction cardiomyopathy. Int J Cardiol. (2009) 134:366–70. 10.1016/j.ijcard.2008.02.023 18579229

[16] Kalapos A Domsik P Forster T Nemes A. Left ventricular strain reduction is not confined to the non-compacted segments in non-compaction cardiomyopathy-insights from the three-dimensional speckle tracking echocardiographic MAGYAR-Path Study. Echocardiography. (2014) 31:638–43. 10.1111/echo.12447 24400635

[17] van Dalen BM Caliskan K Soliman OI Nemes A Vletter WB Ten Cate FJ. Left ventricular solid body rotation in non-compaction cardiomyopathy: a potential new objective and quantitative functional diagnostic criterion? Eur J Heart Fail. (2008) 10:1088–93. 10.1016/j.ejheart.2008.08.006 18815069

[18] Peters F Khandheria BK Libhaber E Maharaj N dos Santos C Matioda H. Left ventricular twist in left ventricular noncompaction. Eur Heart J Cardiovasc Imaging. (2014) 15:48–55. 10.1093/ehjci/jet076 23793875

[19] Nemes A Piros GÁ Domsik P Kalapos A Forster T. Left Atrial Volumetric and Strain Analysis by Three-Dimensional Speckle-Tracking Echocardiography in non-compaction cardiomyopathy: results from the MAGYAR-Path Study. Hellenic J Cardiol. (2016) 57:23–9. 10.1016/S1109-9666(16)30014-8 26856197

[20] Nemes A Anwar AM Caliskan K Soliman OI van Dalen BM Geleijnse ML. Evaluation of left atrial systolic function in non-compaction cardiomyopathy by real-time three-dimensional echocardiography. Int J Cardiovasc Imaging. (2008) 24:237–42. 10.1007/s10554-007-9261-5 17849237 PMC2233707

[21] Nemes A Anwar AM Caliskan K Soliman OI van Dalen BM Geleijnse ML. Non-compaction cardiomyopathy is associated with mitral annulus enlargement and functional impairment: a real-time three-dimensional echocardiographic study. J Heart Valve Dis. (2008) 17:31–5. 18365566

[22] Stämpfli SF Donati TG Hellermann J Anwer S Erhart L Gruner C. Right ventricle and outcome in left ventricular non-compaction cardiomyopathy. J Cardiol. (2020) 75:20–6. 10.1016/j.jjcc.2019.09.003 31587941

[23] Adil A Samad F Bush ML Galazka PZ Tajik AJ. Familial mitral arcade, tricuspid dysplasia, left ventricular non-compaction and short-chain acyl-coa reductase deficiency. Am J Cardiol. (2020) 125:652–7. 10.1016/j.amjcard.2019.11.005 31870493

[24] Tourmousoglou C Bogossian H Ninios V Ninios E. A rare case of Ebstein's anomaly with left ventricular non-compaction. Asian Cardiovasc Thorac Ann. (2019) 27:208–9. 10.1177/0218492318791421 30080103

